# Markers of Early Liver Dysfunction Associate With Reduced Heart Rate Variability in Adolescents With Type 1 Diabetes

**DOI:** 10.1155/pedi/1910554

**Published:** 2025-05-15

**Authors:** Vallimayil Velayutham, Paul Z. Benitez-Aguirre, Gerald Liew, Alicia J. Jenkins, Maria E. Craig, Kim C. Donaghue

**Affiliations:** ^1^Paediatrics, University of Sydney, Sydney, New South Wales, Australia; ^2^Institute of Endocrinology and Diabetes, The Children's Hospital at Westmead, Sydney, New South Wales, Australia; ^3^Ophthalmology, The Children's Hospital Westmead, Sydney, New South Wales, Australia; ^4^Ophthalmology, University of Sydney, Sydney, New South Wales, Australia; ^5^Diabetic and Vascular Medicine, Baker Heart and Diabetes Institute, Melbourne, Victoria, Australia; ^6^NHMRC Clinical Trials Centre, University of Sydney, Sydney, New South Wales, Australia; ^7^Paediatrics, University of New South Wales, Sydney, New South Wales, Australia

## Abstract

**Aim:** Data on the impact of metabolic dysfunction-associated fatty liver disease (MAFLD) on diabetes complications in youth with type 1 diabetes are lacking. However, MAFLD is well known to contribute to cardiovascular disease (CVD) in people with type 2 diabetes. We aimed to investigate markers of MAFLD in youth with type 1 diabetes and their relationship with chronic complications.

**Methods:** A prospective study of 102 adolescents (mean age 14.7 ± 1.9 years) with type 1 diabetes underwent repeated annual diabetes complications assessments, including annual measures of liver enzymes. Early cardiac autonomic nerve dysfunction (CAN) was defined as ≥1 abnormality in seven heart rate variability parameters derived from a 10-min resting electrocardiogram. Multivariate generalized estimating equations explored predictors of CAN and other microvascular complications (retinopathy and early kidney dysfunction).

**Results:** After a median follow-up of 3.5 years (IQR 2.7–4.6), there were significant increases in the mean alanine transaminase level (ALT) and systolic blood pressure (SBP) percentiles. Upper ALT and gamma-glutamyl transferase (GGT) tertiles (T3 vs. T1-2: odds ratio [OR], 95% confidence interval [CI], 2.05 [1.20, 3.48], and 2.99 [1.61, 5.58], respectively) predicted CAN development (23%, *n* = 24) independent of HbA1c and diabetes duration. They were not associated with retinopathy or early kidney dysfunction.

**Conclusion:** Higher ALT and GGT associate with early CAN in adolescents with type 1 diabetes, suggesting hepatic inflammation may compound the impact of the diabetes milieu on systemic endothelial dysfunction.

## 1. Introduction

Nonalcoholic/metabolic dysfunction-associated fatty liver disease (MAFLD) in adults with type 2 diabetes is associated with an increased prevalence of microvascular complications [1], cardiovascular morbidity, and mortality [2]. With the increasing global prevalence of obesity, now affecting more than 340 million children and adolescents [3], metabolic syndrome and MAFLD are not uncommon in type 1 diabetes [4].

There is a paucity of data on MAFLD in children and adolescents with type 1 diabetes. A liver biopsy is a gold standard for diagnosing NAFLD/MAFLD, but this is risky, invasive, and costly. Alanine transaminase level (ALT) is the recommended screening test for diagnosing pediatric NAFLD/MAFLD. The North American Society of Pediatric Gastroenterology, Hepatology and Nutrition (NASPGHAN) recommends sex-specific cut-off values (>22 mg/dL for girls and >26 mg/dL for boys) [5]. However, no consensus ALT threshold is established within the type 1 diabetes population.

We previously demonstrated that higher body mass index (BMI) and obesity in adolescents with type 1 diabetes were associated with a greater risk of cardiac autonomic neuropathy [6]. Although clinically overt CAN is uncommon in type 1 diabetes during childhood and adolescence, subclinical signs are evident within a few years of diabetes onset [7, 8]. Longer diabetes duration, poor glycemic control, and puberty are associated with increased CAN risk [9, 10]. Furthermore, CAN occurs in adults and children with obesity, even without diabetes [11], and significantly increases cardiovascular disease (CVD) risk. We also previously demonstrated CAN predates retinopathy and early kidney dysfunction in adolescents [12, 13]. It is well-recognized that retinal and renal complications are associated with increased CVD risk in people with type 1 diabetes [14].

We hypothesized that liver transaminases are surrogates of MAFLD and predictors of CAN and other microvascular complications in adolescents with type 1 diabetes.

## 2. Research Design and Methods

### 2.1. Study Population

A longitudinal study of 102 adolescents with type 1 diabetes underwent diabetes complications assessments at The Children's Hospital at Westmead in Sydney, Australia, with a minimum of three visits and measures of ALT, gamma-glutamyl transferase (GGT), and aspartate transaminase (AST). Inclusion criteria were age 12–20 years and >2 years of type 1 diabetes duration. Complete case analyses of 102 patients (323 visits) were included to explore associations between liver enzymes and diabetes complications (CAN, retinopathy, and early kidney dysfunction). A subgroup analysis of 77 adolescents without baseline CAN was included in the incident CAN analysis. The Sydney Children's Hospitals Network ethics committee approved the study, and written informed consent/assent was obtained.

### 2.2. Complications Assessment

Participants were assessed at each complication assessment visit by standardized interviews, clinical examinations, and laboratory investigations. Briefly, height and weight were measured to estimate BMI (kg/m^2^), and SD scores were determined using population-based data from the Center for Disease Control (USA). BP was measured with a sphygmomanometer using an appropriately sized cuff in seated individuals after 5 min of rest. Nonfasting venous blood samples were obtained to measure total cholesterol and triglyceride levels, glycemic control (HbA1c), and liver enzymes (ALT, GGT, and AST), which were quantified using a VITROS 5600 (Ortho Diagnostics, USA) autoanalyser. Urinary albumin excretion was measured from three overnight timed urine collections using an Immulite analyzer (Siemens, Los Angeles, CA).

### 2.3. Cardiac Autonomic Nerve Testing

Participants underwent a 10-min continuous ECG recording (LabChart-Pro, ADInstruments, Sydney, Australia) in a supine position after 10 min of rest in a quiet room to assess heart rate variability. The entire 10-min recording was used except for ectopic beats (<500 ms, >1100 ms). All traces were analyzed by a single operator (TJ), who checked for artifacts and ectopic beats. Early CAN was defined as ≥1 abnormal heart rate test out of seven tests: Time-domain derived measures of HRV were: (1) the standard deviation of mean NN intervals (SDNN) (NN used in place of RR and is determined from adjacent QRS-complexes) and root mean squared difference of successive NN-intervals (RMSSD), which are estimates of overall HRV. (2) Frequency domain measures were: low frequency (LF), defined as >0.04 Hz and <0.15 Hz; high frequency (HF) components, defined as >0.15 Hz and <0.4 Hz; and the LF: HF ratio, considered to be an estimate of the relative sympathetic and parasympathetic balance. (3) Geometric-domain analyses were performed using triangular index (TI), the total number of all NN intervals divided by the height of the histogram of all NN intervals measured on a discrete scale of bins of 7.8125 ms (1/128 s). Normal reference ranges were derived from HRV measured using the same device on contemporary local controls (healthy school-aged adolescents) without diabetes. The normal range was defined as above the 5^th^ percentile for age and gender for HRV parameters log10 (SDNN), log10 (RMSSD), log10 (TI), and below the 95^th^ percentile for age and gender for heart rate and log10 (LF:HF ratio) [15].

### 2.4. Retinopathy

Mydriatic seven-field stereoscopic fundal photography of both eyes was performed using a TRC 50-VT Topcon Fundus Camera (Tokyo Optical, Tokyo, Japan) to assess retinopathy [16]. Camera settings, including the angle of retinal photography, remained unchanged.

Diabetic retinopathy (DR) was defined as having ≥1 microaneurysm or hemorrhage from seven standard fields. We used the International Clinical Classification of DR, which has five stages based on the Early Treatment of DR Study (ETDRS) modified Airlie House classification.

### 2.5. Early Kidney Dysfunction

Early kidney dysfunction was defined as mean urinary ACR > 3 mg/mmol/min from three overnight timed urine collections, and albuminuria was defined as ACR > 30 mg/mmol.

### 2.6. Statistical Analysis

Descriptive statistics are reported as mean ± SD for normally distributed data or median (interquartile range [IQR]) for non-normally distributed data (e.g., follow-up duration). Participant characteristics were described based on baseline CAN status. Differences in categorical variables were compared using the *χ*^2^ test. Differences in continuous variables between adolescents with and without CAN used analysis of variance (ANOVA). Generalized estimating equations (GEEs) were utilized to explore ALT, GGT, and AST as predictors of CAN, retinopathy, and albumin excretion rate (AER).

Longitudinal analyses were performed using GEE, with the presence or absence of CAN at each visit as the outcome variable. GEE allowed all visits for each participant to be included in the analysis and accounted for correlations between repeated observations for a given participant. Liver enzymes (ALT, GGT, and AST) were first examined as continuous variables and then grouped into tertiles to investigate potential threshold effects. Diabetes duration, HbA1c, and tertiles of ALT, GGT, and AST were included in the multivariable model as covariates. Only visits with all covariates were used in the final model. Adolescents with baseline CAN were excluded (*n* = 25, 24%) to explore the predictors of incident CAN. GEE results are expressed as odds ratio (OR) and 95% confidence interval (CI).

Cox proportional hazard regression analysis was used to explore the threshold effects of liver enzymes (ALT, GGT, and AST) on the cumulative risk of CAN. We used tertiles of liver enzymes and included HbA1c and diabetes duration in the Cox regression analysis. Analyses were performed using SPSS (Version 26, IBM Corp, 2018), and statistical significance was taken at *p* < 0.05.

## 3. Results

At baseline, 102 participants (49 male), the mean age was 14.7 ± 1.9 years; diabetes duration was 7.5 ± 3.2 years, BMI standard deviation score (SDS) was 0.71 ± 0.87, and HbA1c IFCC 66.6 ± 15.9 mmol/mol (8.2 ± 1.4%) (Table 1). When examined by the presence or absence of CAN at the first visit, adolescents with CAN had higher ALT levels (18.6 ± 10.1 vs. 15.1 ± 7.6; *p*=0.049). There were no significant differences in age, diabetes duration, HbA1c, GGT, or AST (Table 1). Adolescents with CAN and overweight/obesity (BMI > 85^th^ centile) had higher ALT (21.3 IU/L vs. 14.5 IU/L *p*=0.03) and AST (26.5 IU/L vs. 22.2 IU/L, *p*=0.004) compared to adolescents without CAN and higher BMI.

**Figure 1:**
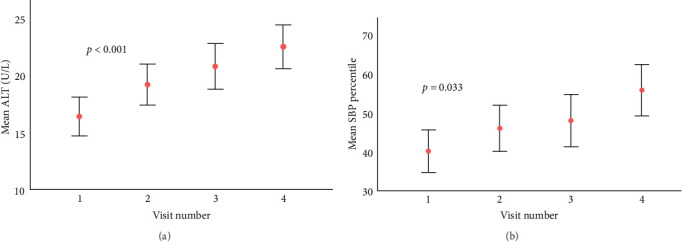
Progressive increase in ALT levels and systolic blood pressure during the study period. (A) It shows a progressive increase in ALT. (B) It shows an increase in the SBP percentile. Aspartate transaminase (AST), gamma-glutamyl transferase (GGT), BMI, and diastolic blood pressure did not show progressive increases during the study period. The analysis included all 102 participants and 323 visits.

After a median follow-up of 3.5 years (IQR 2.7–4.6), there were significant increases in ALT levels and systolic blood pressure (SBP) percentiles (Figure 1). Those in the upper ALT tertile (>22 IU/L) had higher SBP percentile (51^st^ centile vs. 44^th^ centile, *p*=0.03) and higher TG levels (mean 1.22 mmol/L vs. 1.05 mmol/L, *p*=0.02). Similarly, those in the upper GGT tertile (>15 IU/L) had higher TG (1.27 vs. 1.04 mmol/L, *p*=0.001), higher HbA1c (73 vs. 67 mmol/mol, 8.8% vs. 8.3%, *p*=0.006) and longer diabetes duration (10 vs. 9 years, *p*=0.004).

**Figure 2:**
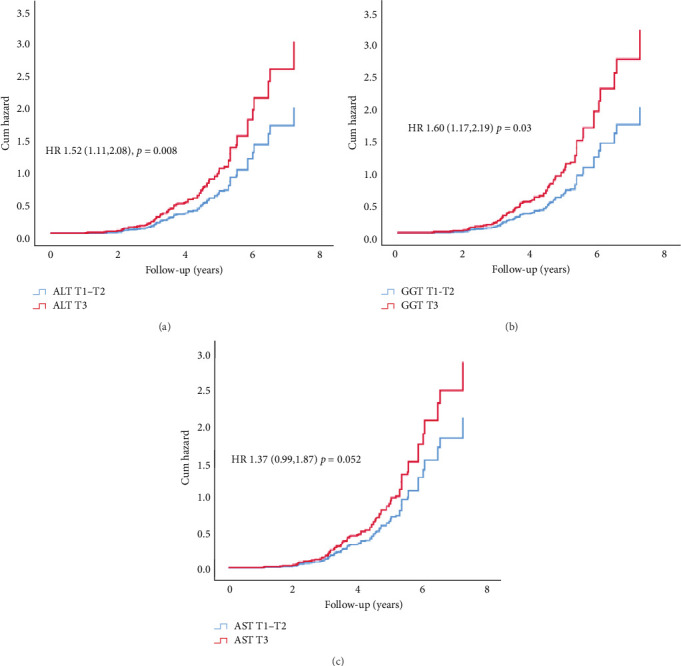
Cumulative hazard curves for risk of early cardiac autonomic neuropathy. (A) Cumulative hazard curves for risk of CAN by ALT tertiles: tertile 3 vs tertiles 1–2 (HR 1.52 95% CI [1.11, 2.08]) adjusted for HbA1C and duration. (B) Cumulative hazard curves for risk of CAN by GGT tertiles: tertile 3 vs tertiles 1–2 (HR 1.60 [1.17, 2.19]) adjusted for HbA1C and duration. (C) Cumulative hazard curves for risk of CAN by AST tertiles: tertile 3 vs tertiles 1–2 (HR 1.37 [0.99, 1.87]) adjusted for HbA1C and duration.

In Cox proportional hazard regression analysis, upper tertile ALT and GGT were associated with increased risk of CAN (ALT: hazard ratio [HR] 1.52, 95% CI [1.12, 2.07] and GGT 1.60 [1.17, 2.19], respectively), independent of HbA1c and diabetes duration (Figure 2).

### 3.1. Incident CAN Subgroup

In univariable GEE analysis (77 participants, 246 visits), upper tertiles (T3 vs. T1-2) of ALT and GGT (OR 2.09, 95% CI [1.23, 3.57] and 3.08 [1.66,5.73], respectively) predicted incident CAN. ALT as a continuous variable predicted incident CAN (1.04 [1.02,1.07]), whilst AST and GGT did not reach significance. Longer diabetes duration and higher HbA1c levels were associated with greater risk of incident CAN (diabetes duration: 1.23, 95% [1.12,1.35] and HbA1c: 1.18 [1.003,1.40]). Blood pressure percentiles, triglyceride levels, mean ACR, BMI/overweight, and obesity were not significantly associated with incident CAN.

In multivariable GEE analysis, upper tertile ALT and GGT predicted incident CAN (OR ALT 2.09 [1.23,3.57] and GGT 2.99 [1.61,5.58]) independent of diabetes duration and HbA1c (Table 2). Higher GGT tertile predicted retinopathy in univariable analysis (OR 2.16, 95% CI [1.08, 4.31]). In multivariable analysis, adjusting for HbA1c and diabetes duration, this association did not reach significance. AST and ALT were not significant predictors of retinopathy or early kidney dysfunction.

## 4. Discussion

In this longitudinal study, we demonstrate that higher ALT and GGT tertiles are associated with early CAN, suggesting their utility as markers of metabolic dysfunction and subsequent cardiovascular risk in adolescents with type 1 diabetes.

Incident early CAN abnormalities developed in 23% of participants, consistent with previous studies. In youth with type 1 diabetes, the prevalence of cardiac autonomic neuropathy is highly variable due to the testing methods, diagnostic criteria, population, and age-matched normal values [15, 17–21]. The SEARCH study utilized 3 abnormal HRV tests to define CAN and reported a prevalence of 12% [18]. In an earlier metanalysis, the pooled prevalence of CAN in youth was found to be 21% determined by heart rate variability testing, and 28% determined from all studies [22]. In the current study, ALT increased over time and, when examined both continuously and categorically (upper tertile ALT), was associated with important features of metabolic syndrome, namely higher TG levels and SBP centiles. AST and GGT did not increase over time in our study and are generally considered to have poor sensitivity for MAFLD. However, in children with high ALT levels, elevated AST and GGT levels are associated with worse MAFLD histology [23].

Overweight and obesity were observed in 37% of our cohort at baseline. The prevalence of being overweight or obese in this cohort is consistent with other diabetes groups and the general population. Notably, BMI was not associated with CAN or ALT levels. Data suggests that waist circumference may better indicate central adiposity, insulin sensitivity, and metabolic syndrome [6], which may be reflected in ALT levels. We observed that adolescents with CAN had a greater waist circumference at baseline, in keeping with previous studies demonstrating central adiposity as a predictor of CAN [6]. However, waist circumference data were limited and available in <70% of visits in our study.

Inflammation, insulin resistance, and hepatic triglyceride accumulation play a major role in MAFLD. Visceral fat promotes increased levels of plasminogen activator inhibitor 1 (PAI-1) and vascular endothelial growth factor (VEGF), which are associated with increased risk of DR [24–26]. Inflammatory and insulin-resistant states ensue due to the increased production of inflammatory cytokines from visceral fat, which in turn increases triglyceride levels [27, 28].

Metabolic syndrome is an important risk factor for the development of CAN [6, 29]. Auto-regulatory dysfunction in the liver microvasculature may occur concurrently with microvasculature dysfunction in the myocardium. Chronic hyperglycemia leads to progressive autonomic dysfunction, resulting in increased sympathetic tone and subsequent mitochondrial oxidative stress, leading to CAN. Endothelial dysfunction in diabetes and insulin resistance/metabolic syndrome secondary to obesity can further exaggerate myocardial perfusion defects [30]. Evidence is mounting that myocardial microvascular dysfunction, resulting in impaired myocardial blood flow and perfusion defects [31], develops early in diabetes and contributes to increased cardiovascular risk [32].

To our knowledge, this is the first longitudinal study in adolescents with type 1 diabetes describing the association between markers of MAFLD and CAN. In our study, liver enzymes were not associated with retinopathy or early kidney dysfunction, however, this is likely due to the relatively small number of participants and warrants further investigation. We propose a chronology of events in adolescents with type 1 diabetes whereby a systemic diabetic endotheliopathy results in early liver dysfunction and a proinflammatory state. This contributes to autonomic dysregulation, the development of CAN, and subsequently other microvascular complications [12].

Ultrasound of the liver is not sensitive or specific, especially in obesity and when steatosis is present in less than 33% of hepatocytes [33], and more sensitive imaging was not available. Quantitative ultrasound, MRI, MRI protein density fat fraction (MRI-PDFF), and magnetic resonance spectroscopy (MRS) help in the accurate diagnosis of MAFLD [34, 35]. FibroScan is a noninvasive method for diagnosing MAFLD, but normative data for children and adolescents are not available [36]. Various factors, including increased BMI, limit the accuracy [37]. However, these modalities are expensive and not widely available.

The strengths of our study are the novel investigation of repeated screening measures for NAFLD/MAFLD in this age group. Limitations include the sample size and utilization of a single test abnormality to define early CAN, serum biomarkers rather than radiological investigation, including FibroScan or biopsy, to diagnose liver pathology. A liver biopsy is the gold standard for diagnosing MAFLD, but it has risks and costs. Noninvasive screening tools include ultrasound, but this has low sensitivity and specificity.

Current guidelines do not include recommendations for screening for MAFLD in type 1 diabetes. ISPAD 2024 guidelines for type diabetes recommend liver enzymes as a part of complication screening in type 2 diabetes. ADA guidelines recommend screening with liver transaminases in type 2 diabetes and consider screening in type 1 diabetes with risk factors, such as obesity or incidental finding of elevated transaminases or hepatic steatosis in imaging. Findings from this study provide evidence that liver transaminases can be affected early in the disease process. Liver function testing could be included in complication screening in type 1 diabetes.

In this study, we demonstrated that higher ALT and GGT levels are associated with abnormal heart variability in adolescents with type 1 diabetes. These findings likely reflect the compounding impact of hepatic inflammation on systemic and endothelial dysfunction in the diabetic milieu. Future studies detailed study involving a larger group of patients, glycaemic variability from continuous glucose monitoring, and longer follow-ups are merited.

## Figures and Tables

**Table 1:** Patient characteristics stratified by CAN status at baseline.

Characteristic	All *N* = 102	Presence of CAN *N* = 25	No CAN *N* = 77	*p*
Age (years)	14.7 ± 1.9	14.6 ± 1.8	14.7 ± 1.9	0.838
HbA1C (mmol/mmol)	66.6 ± 15.9	71.6 ± 19.4	64.8 ± 14.2	0.303
HbA1C (%)	8.2 ± 1.4	8.5 ± 1.6	8.1 ± 1.3	0.303
Duration (years)	7.5 ± 3.2	6.7 ± 2.8	7.9 ± 3.3	0.087
SBP SDS	−0.30 ± 0.94	−0.09 ± 1.03	−0.41 ± 0.88	0.120
DBP SDS	0.06 ± 0.80	0.15 ± 0.73	0.02 ± 0.73	0.424
BMI SDS	0.71 ± 0.87	0.85 ± 0.76	0.64 ± 0.91	0.248
Percentage of overweight and obesity (%)	37% (38/102)	60% (15/25)	29% (22/77)	0.548
Waist circumference (cm)^a^	75.9 ± 10.4	77.1 ± 9.7	75.3 ± 10.8	0.556
ALT (U/L)^b^	16.3 ± 8.6	18.6 ± 10.1	15.1 ± 7.6	0.049
AST (U/L)	24.4 ± 5.7	25.6 ± 5.9	24.0 ± 5.5	0.110
GGT (U/L)	13.6 ± 5.6	14.2 ± 5.9	13.4 ± 5.9	0.513
Total cholesterol (mmol/L)	4.5 ± 0.82	4.7 ± 0.81	4.5 ± 0.83	0.256
HDL-C (mmol/L)	1.44 ± 0.30	1.42 ± 0.25	1.45 ± 0.32	0.729
TG (mmol/L)	1.14 ± 0.66	1.21 ± 0.82	1.09 ± 0.57	0.412
Mean ACR (mg/mmol)	1.05 ± 1.14	1.02 ± 1.18	1.07 ± 1.13	0.845
Retinopathy	11	5 (20%)	6 (9%)	0.678
DPN	19	8 (32%)	11 (14%)	0.571
Microalbuminuria	4	2 (8%)	2 (2.6%)	0.407

*Note*: Retinopathy, presence of one or microaneurysms.

Abbreviations: ACR, albumin creatinine ratio; ALT, alanine transaminase; AST, aspartate transaminase; BMI, body mass index; DPN, distal peripheral neuropathy; GGT, gamma glutamyl transferase; TG, triglyceride.

^a^Data available for 53 participants.

^b^ALT was significantly higher in adolescents with CAN (*p* < 0.05) compared with no CAN.

**Table 2:** Predictors of incident cardiac autonomic nerve abnormalities.

Characteristic	Univariate	*p*	Multivariable	*p*
Model 1				
ALT tertile 3 vs	2.09 (1.23, 3.57)	0.007	2.05 (1.20, 3.48)	0.008
tertile1–2	Ref	—	Ref	—
Duration	1.23 (1.12, 1.35)	<0.001	1.03 (0.95, 1.11)	0.471
HbA1C	1.18 (1.003, 1.40)	0.046	1.11 (0.89, 1.37)	0.341
Model 2				
GGT tertile 3 vs	3.08 (1.66, 5.73)	<0.001	2.99 (1.61, 5.58)	0.001
tertile1–2	Ref	—	Ref	—
Duration	1.23 (1.12, 1.35)	<0.001	1.01 (0.93, 1.09)	0.861
HbA1C	1.18 (1.003, 1.40)	0.046	1.11 (0.90, 1.37)	0.319
Model 3				
AST tertile 3 vs	1.08 (0.57, 2.07)	0.797	1.14 (0.59, 2.20)	0.689
tertile1–2	Ref	—	Ref	—
Duration	1.23 (1.12, 1.35)	<0.001	1.03 (0.95, 1.12)	0.419
HbA1C	1.18 (1.003, 1.40)	0.046	1.12 (0.91, 1.39)	0.287

*Note*: Analysis included 77 adolescents with 246 visits.

## Data Availability

The data that support the findings of this study are available from the corresponding author upon reasonable request.

## Nomenclature

ALT: Alanine transaminase
AST: Aspartate transaminase
AER: Albumin excretion rate
BMI: Body mass index
CAN: Cardiac autonomic nerve dysfunction
CI: Confidence interval
DBP: Diastolic blood pressure
GGT: Gamma glutamyl transferase
GFR: Glomerular filtration rate
GEE: Generalized estimating equation
HR: Hazard ratio
OR: Odds ratio
SBP: Systolic blood pressure
SDS: Standard deviation score.
